# Intracellular localization of vitellogenin receptor mRNA and protein during oogenesis of a parthenogenetic tick, *Haemaphysalis longicornis*

**DOI:** 10.1186/s13071-019-3469-9

**Published:** 2019-05-06

**Authors:** Rika Umemiya-Shirafuji, Ryo Mihara, Kozo Fujisaki, Hiroshi Suzuki

**Affiliations:** 1 National Research Center for Protozoan Diseases, Obihiro University of Agriculture and Veterinary Medicine, Inada-Cho, Obihiro, Hokkaido 080-8555 Japan; 2 National Agricultural and Food Research Organization, Kannondai 3-1-5, Tsukuba, Ibaraki 305-0856 Japan

**Keywords:** Tick, *Haemaphysalis longicornis*, Vitellogenin receptor, Ovary, Oocyte

## Abstract

**Background:**

Vitellogenin (Vg), a key molecule for oocyte development synthesized in the fat body during blood-feeding, is released into the hemolymph and then taken into the oocytes *via* Vg receptor (VgR) in ticks. Previously, we showed that *VgR* mRNA is expressed in the ovary at the adult stage of parthenogenetic *Haemaphysalis longicornis* ticks and its expression increases after blood-feeding. However, intracellular localization of VgR mRNA and protein at each developmental stage of oocytes during oogenesis remains largely unclear.

**Methods:**

mRNA and protein expression profiles of *H. longicornis* VgR (HlVgR) in the oocytes from the unfed to oviposition periods were analyzed by real-time PCR, *in situ* hybridization, and immunostaining. To elucidate the timing of the onset of Vg uptake, RNA interference (RNAi)-mediated gene silencing of *HlVgR* was performed.

**Results:**

*In situ* hybridization revealed that *HlVgR* mRNA was detected in the cytoplasm of stage I-III oocytes, and weaker positive signals for *HlVgR* mRNA were found in the cell periphery of stage IV and V oocytes. Likewise, HlVgR protein was detected by immunostaining in the cytoplasm of stage I-III oocytes and in the cell periphery of stage IV and V oocytes. Each developmental stage of the oocytes showed distinct patterns of mRNA and protein expression of HlVgR. Moreover, RNAi of *HlVgR* caused delayed or arrested development in the oocytes. The ovaries of control ticks showed all developmental stages of oocytes, whereas stage I-III oocytes were found in the ovaries of *HlVgR*-RNAi ticks at 5 days after engorgement.

**Conclusions:**

These results suggest that active uptake of Vg is required for development from stage III to stage IV during oogenesis. Our data clearly revealed an apparent shift in the intracellular localization of VgR for both mRNA and protein level in oocytes during oogenesis.

## Background

Ticks, vectors of harmful pathogens for human and animals, absolutely require a blood meal for their development and reproduction. Nutrients derived from a blood meal allow the synthesis of vitellogenin (Vg), an essential molecule for oocyte development, in the fat body of female ticks, being stimulated by ecdysteroid hormones [1–10]. The regulatory network underlying the synthesis of Vg (vitellogenesis) in ticks is being clarified by RNA interference (RNAi)-mediated gene knockdown experiments and inhibitor assays. A GATA factor was found to be a transcriptional activator of the *Vg* gene in the female *Haemaphysalis longicornis* [11]. Subsequent studies revealed that vitellogenesis is controlled by the activation of the target of rapamycin (TOR), a key molecule of a nutrient-sensing pathway in eukaryotic cells, through ecdysteroid hormone stimulation [12]. TOR phosphorylates S6 kinase and regulates the transcription and intracellular localization of a GATA factor in the fat body. Our previous data suggested that the serine/threonine protein kinase Akt is an upstream factor of TOR and essential not only for controlling gene transcription but also for regulating vitellogenesis [13].

Following vitellogenesis, Vg is released into the hemocoel filled with hemolymph and then is taken into developing oocytes in ticks [7, 10]. The absorption of Vg into the oocytes occurs *via* receptor-mediated endocytosis, and the Vg receptor (VgR) is a member of the low-density lipoprotein receptor (LDLR) superfamily [14]. In hard ticks, homologues of VgR have been identified from *Amblyomma hebraeum* (*AhVgR*) [15], *Dermacentor variabilis* (*DvVgR*) [16], *H. longicornis* (*HlVgR*) [17], *Rhipicephalus microplus* (*RmVgR*) and *R. appendiculatus* (*RaVgR*) [18]. These tick VgRs have common structural elements similar to those in insects: LDLR class A repeats, LDLR class B repeats, epidermal growth factor (EGF) precursor homology domains containing YWXD repeats, an O-linked carbohydrate domain, a transmembrane domain and a cytoplasmic domain [14–18]. The predicted molecular weight of tick VgR based on the amino acid sequences is approximately 200 kDa [15–18]. These studies showed that knockdown of *VgR* in female ticks led to the failure of Vg uptake in the oocytes and the subsequent oviposition, even though the ticks engorged completely. Immunoelectron microscopy revealed that VgR is localized on the external surface of the plasma membrane in developing tick oocytes [17]. In addition, whole tissue *in situ* hybridization using the ovaries of engorged females at 10 days following engorgement revealed that *VgR* mRNA was detected in the smallest oocytes, which had not yet begun to fill with yolk particles [15]. However, little is known about intracellular localization of VgR mRNA and protein at each developmental stage, namely stages I to V, of oocytes throughout oogenesis.

Transmission of some pathogens is tightly linked to the reproduction system in female ticks. After ingesting a blood meal containing some pathogens, ticks may give rise to progeny with these pathogens. An ixodid female tick generally lays a few thousand eggs [19]; therefore, the basis of oogenesis is an important key for the development of novel control strategies for pathogen transmission by ticks. For instance, it has been reported that VgR might be involved in transovarial transmission of *Babesia* parasites by a vector tick *H. longicornis* [17]. This finding led to the hypothesis that *Babesia* parasites bind to tick VgR and invade the developing oocytes of ticks and that some surface molecules of parasites have ligand-binding activity for VgR. Therefore, it is important to determine the expression pattern and intracellular localization of VgR to elucidate the interactions between *Babesia* parasites and tick VgR during oogenesis.

In the present study, we examined mRNA and protein expression profiles of HlVgR in the oocytes from the unfed to the oviposition periods by real-time PCR and *in situ* hybridization and immunostaining using ovarian tissue sections. Furthermore, to determine the timing of the onset of Vg uptake during oogenesis, oocytes of *HlVgR*-RNAi ticks were observed histologically on the basis of the classification criteria [20] for developing oocytes in the parthenogenetic *H. longicornis*.

## Results

### Expression of *HlVgR* mRNA in the ovary

**Fig. 1:**
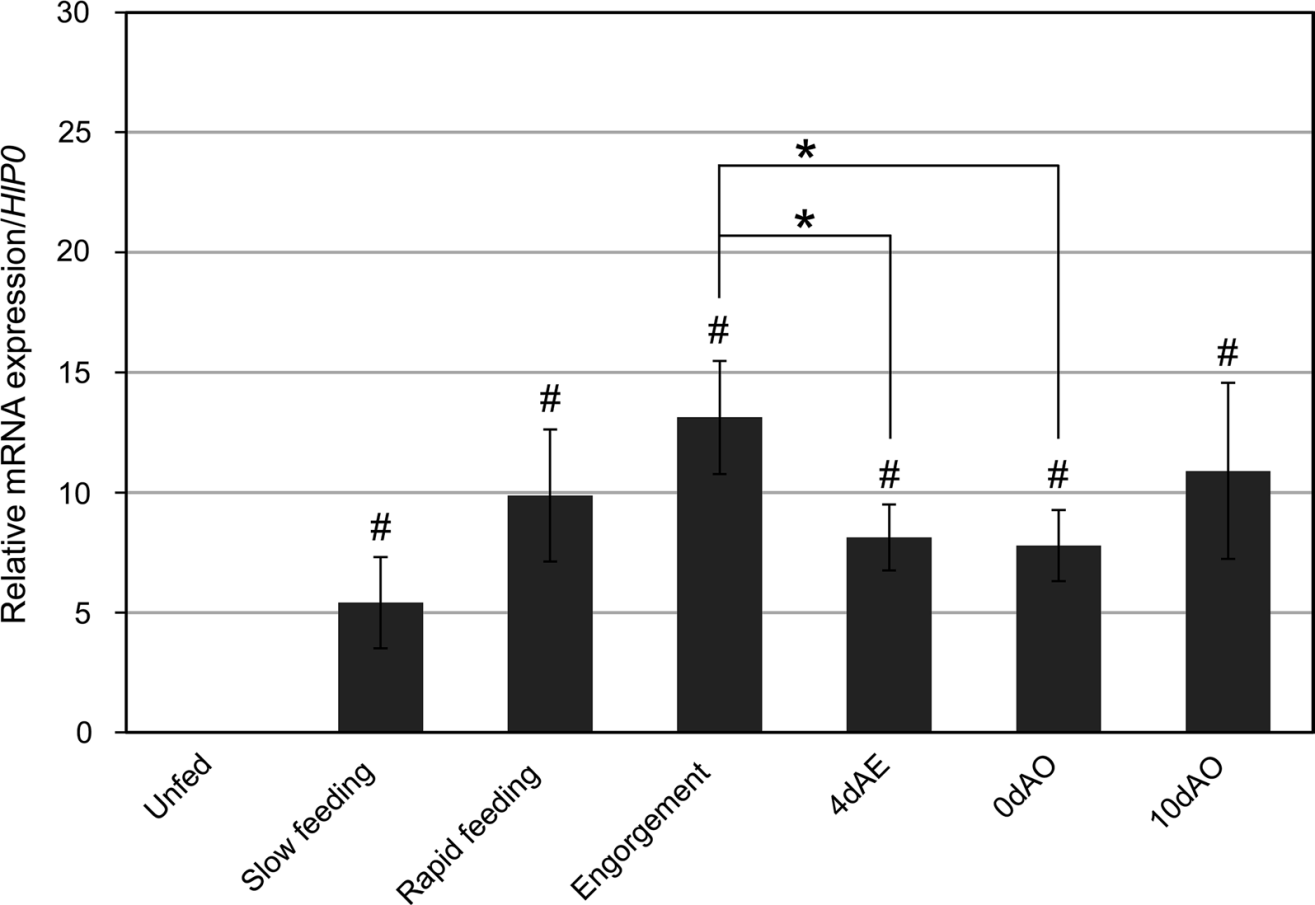
mRNA expression of *HlVgR* in the ovaries of *H. longicornis* from unfed to oviposition periods. Total RNA was extracted from each ovary sample. DNase-treated RNA was subjected to cDNA synthesis. Data were normalized by a real-time PCR analysis of *HlP0* mRNA levels in the cDNA samples. Data represent the mean ± SD of triplicate samples. Asterisk and hash symbols indicate significant downregulation (Student t-test) and upregulation (Welchʼs t-test), respectively, at *P* < 0.01. *Abbreviations*: 4dAE, 4 days after engorgement; 0dAO, the beginning of oviposition; 10dAO, 10 days after the beginning of oviposition

Real-time PCR with specific primers was conducted to determine the relative expression level of *HlVgR* in the ovary from the unfed to the oviposition periods. Average value of *HlVgR* mRNA expression was 0.005 in the unfed period. By contrast, the expression at the slow feeding was more than 1000-fold higher than that at the unfed period (*t*_(5)_ = − 6.957, *P* = 0.0009) (Fig. 1). The expression levels of *HlVgR* in the rapid feeding period were higher than those at previous periods (*t*_(5)_ = − 8.798, *P* = 0.0003) and were the highest at engorgement (*t*_(5)_ = − 13.761, *P* < 0.0001). These results suggest that *HlVgR* was upregulated in the ovary during blood-feeding. In addition, expression levels of *HlVgR* at 4 days after engorgement (4dAE) (*t*_(10)_ = 4.487, *P* = 0.0012) and at the beginning of the oviposition period (0dAO) (*t*_(10)_ = 4.707, *P* = 0.0008) decreased significantly compare with those at engorgement. At 10 days after the beginning of oviposition (10dAO), *HlVgR* expression appeared to be increased again.

### Localization of *HlVgR* mRNA in the ovary

**Fig. 2:**
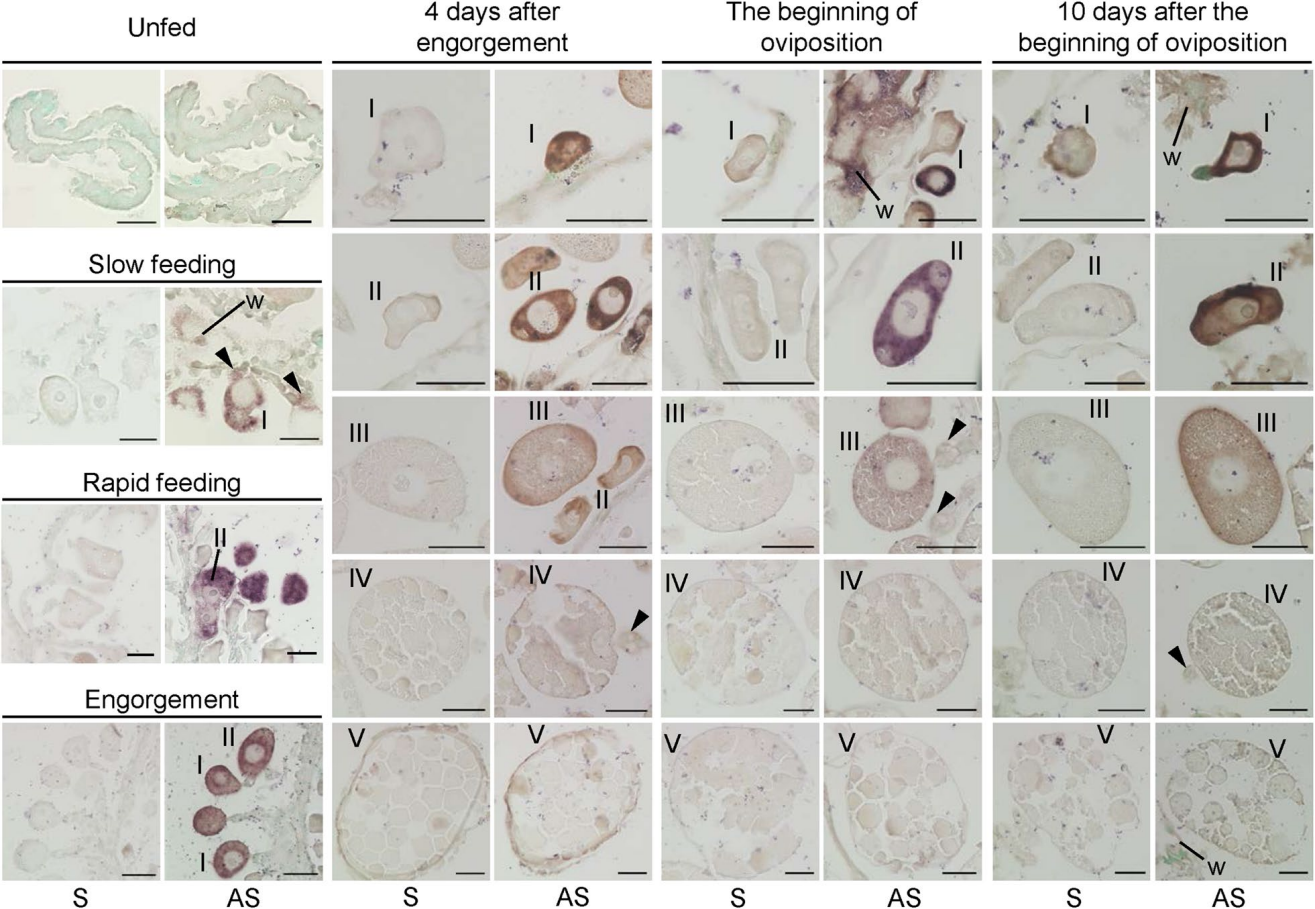
Expression sites of *HlVgR* mRNA in *H. longicornis* ovary from unfed to oviposition periods. Paraffin sections of the ovaries were treated with sense probe (S) or antisense probe (AS). Arrowheads show pedicel cells. *Abbreviations*: W, ovarian wall; I, stage I oocyte; II, stage II oocyte; III, stage III oocyte; IV, stage IV oocyte; V, stage V oocyte. *Scale-bars*: 50 µm

*HlVgR* mRNA was undetected by *in situ* hybridization in the ovaries in the unfed period (Fig. 2). The cytoplasm of oocytes at stages I and II was stained with the antisense probe of *HlVgR* in the slow feeding, rapid feeding, and engorgement periods. The expression of *HlVgR* mRNA in oocytes of all stages (stages I to V) was detected at 4dAE, 0dAO, and 10dAO. *HlVgR* mRNA was localized throughout the cytoplasm in the oocytes of stages I to III. Positive signals for *HlVgR* mRNA were found in the external region of the oocytes that were at stages IV and V, but the signals seemed to be weaker than those of the former stages. The expression of *HlVgR* mRNA was also detected in pedicel cells and the ovarian wall (Fig. 2).

### Localization of HlVgR protein in the ovary

**Fig. 3:**
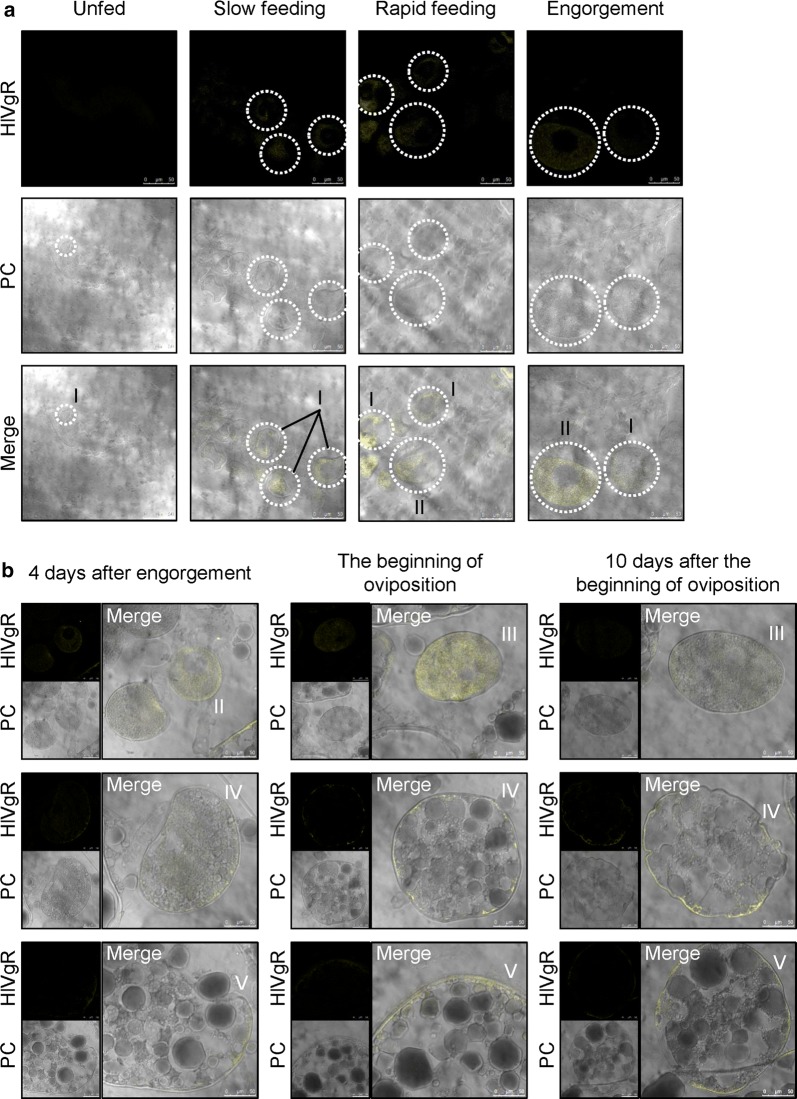
Localization of HlVgR protein in the ovary of *H. longicornis*. Positive signals are displayed in yellow color in the ovarian sections from unfed to engorgement periods (**a**) and from 4 days after engorgement to oviposition periods (**b**). Stage I or II oocytes at unfed to engorgement periods were observed in areas surrounded by dashed circles. *Abbreviations*: PC, phase contrast; I, stage I oocyte; II, stage II oocyte; III, stage III oocyte; IV, stage IV oocyte; V, stage V oocyte. *Scale-bars*: 50 µm

Positive signals of HlVgR were not detected by immunofluorescence staining in the ovary in the unfed period (Fig. 3a) but were found in the cytoplasm of oocytes in the slow feeding period. In the rapid feeding and engorgement periods, HlVgR signals were detected throughout the cytoplasm of stage I and II oocytes. The HlVgR signals were also detected in stage II to V oocytes at 4dAE, 0dAO, and 10dAO (Fig. 3b). HlVgR signals were scarcely observed in stage I oocytes during these periods. HlVgR signals were detected throughout the cytoplasm of the oocytes of stage II. Notably, the signals were localized throughout the cytoplasm as well as external region of the stage III oocytes, whereas the signals were detected at the chorion and the cell periphery in the oocytes of stages IV and V. In addition, HlVgR signals were localized in the cytoplasm surrounding the yolk granules. HlVgR signals were not detected in the pedicel cells and ovarian wall at any period examined in our study.

### Oocyte development in *HlVgR*-RNAi ticks

**Fig. 4:**
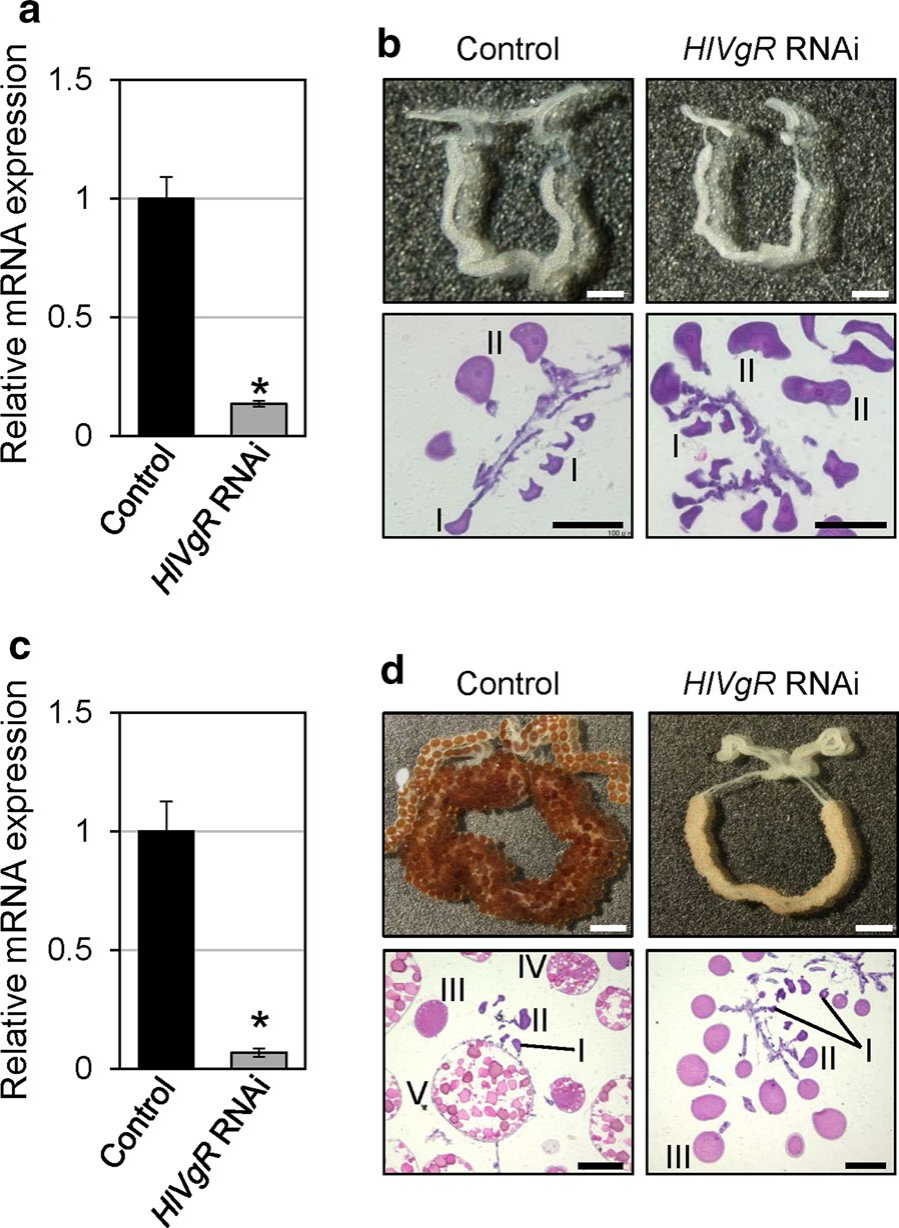
*HlVgR*-knockdown by RNA interference (RNAi) in *H. longicornis*. **a**, **c** Total RNA was purified from engorged ticks in each group. The gene silencing of *HlVgR* was assessed by real-time PCR. The *HlVgR* expression was normalized to the *HlP0* mRNA expression. Data represent the mean ± SD of triplicate samples. *HlVgR* mRNA expressions of RNAi-treated ticks (*HlVgR* RNAi) were significantly lower than those of control ticks (Control) at both engorgement (**a**) and at 5 days after engorgement (**c**) (*P < 0.01; Welchʼs t-test). **b**, **d** Phenotypes of the ovaries of *HlVgR* RNAi-treated and control ticks. Note that *HlVgR*-knockdown caused delayed development in the oocytes at both engorgement (**b**) and at 5 days after engorgement (**d**). *Abbreviations*: I, stage I oocyte; II, stage II oocyte; III, stage III oocyte; IV, stage IV oocyte; V, stage V oocyte. *Scale-bars*: **b**, 1 mm for ovaries and 100 µm for ovarian sections; **d**, 2 mm for ovaries, and 200 µm for ovarian sections

To clarify the timing of the onset of Vg uptake during oogenesis, oocytes in the ovaries were observed after RNAi of the *HlVgR* gene. Real-time PCR revealed decreased expression levels of *HlVgR* mRNA at engorgement (*t*_(2)_ = 16.327, *P* = 0.0037) and at 5 days after engorgement (*t*_(2)_ = 12.749, *P* = 0.0061) compared with that in the control ticks (Fig. 4a, c). There was no difference in the body weight of engorged ticks between the *HlVgR*-RNAi and control groups (Table 1). The oocytes were typically colorless and white in color in both the *HlVgR*-RNAi and control ticks at engorgement (Fig. 4b). At this period, oocytes of stages I and II were observed in both ovaries on the histological sections. Control ticks had amber-colored oocytes in the ovaries and began oviposition at 5 days after engorgement, whereas the ovaries in the *HlVgR*-RNAi ticks possessed amberlite and white colored oocytes, which were classified into stages I to III (Fig. 4d). No yolk granules were found in the cytoplasm of any of the oocytes of the *HlVgR*-RNAi ticks. Although *HlVgR*-RNAi ticks began oviposition at 12 days after engorgement (Table 1), the eggs were not in amber color and not coated with waxy components as shown previously [17].

**Table 1:** Effects of *HlVgR*-knockdown by RNA interference (RNAi) in oocyte development of *H. longicornis*

Groups	No. of ticks	Body weight of engorged ticks (mg)	Typical color of oocytes		Developmental stages of oocytes		Onset of oviposition
			E	5dAE	E	5dAE	
Control	20	239.0 ± 45.7	Colorless and white	Amber	Stages I–II	Stages I–V	At 5 days after engorgement
*HlVgR* RNAi	15	208.3 ± 52.4	Colorless and white	Amberlite and white	Stages I–II	Stages I–III	At 12 days after engorgement

*Abbreviations*: E, engorgement; 5dAE, 5 days after engorgement

## Discussion

Expression levels of *HlVgR* mRNA in the ovary of *H. longicornis* increased with the progress of blood-feeding and reached the highest level at engorgement, suggesting that blood-feeding is a triggering process for the upregulation of *HlVgR* mRNA (Fig. 1). Because ticks have a panoistic ovary, the ratio of each oocyte stage is asynchronous during oogenesis [20]. Subsequently, the spatial patterning of *HlVgR* mRNA expression in each developmental stage of the oocytes was examined by *in situ* hybridization. The cytoplasm of oocytes at stages I and II was intensely stained with the antisense probe of *HlVgR* at the slow feeding, rapid feeding, and engorgement periods (Fig. 2). At these periods, the ovaries of parthenogenetic *H. longicornis* possess stage I and II oocytes, and the ratio of stage II to all oocytes in the rapid feeding period increased at engorgement [20]. In addition, the *HlVgR* mRNA level was significantly decreased at 4dAE and at 0dAO compared with that at engorgement (Fig. 1). At 4dAE and 0dAO, more than half of all oocytes were classified into stages III, IV or V [20]. By linking mRNA expression patterns with morphological features of oocytes, it was obvious that *HlVgR* was highly expressed in stage I and II oocytes compared to the other stages (Figs. 1 and 2 and [20]). These findings substantially correspond with the findings that a high degree of positive staining was detected in the smallest oocytes of *A. hebraeum* by whole tissue *in situ* hybridization [15]. Moreover, *HlVgR* mRNA was detected in the whole cytoplasm of stage I to III oocytes and under the oocyte membrane of oocytes at stages IV and V in our study (Fig. 2). The positive signals of *HlVgR* mRNA in mature oocytes seemed to be weaker than those of stage I-III oocytes. Taken together, real-time PCR and *in situ* hybridization showed that the transcription of *HlVgR* was activated in stages I and II of the oocytes, namely before the formation of yolk granules, regardless of the number of days after blood-feeding.

Immunostaining revealed that HlVgR was localized throughout the cytoplasm of the stage I–III oocytes and in the peripheral region of the stage IV and V oocytes (Fig. 3). As with the results of *in situ* hybridization, the intracellular localization of HlVgR changed as oocyte development progressed. *Drosophila melanogaster* VgR is uniformly distributed throughout the oocyte during the pre-vitellogenic stages of oogenesis, indicating that such oocytes do not take in Vg in the presence of VgR [21]. After the transition to the vitellogenic stages, VgR levels increase markedly at the cell periphery in the oocyte. By the end of vitellogenesis, the receptor localizes predominantly in the external region of the oocyte in the fruit fly. In the yellow fever mosquito *Aedes aegypti*, VgR is internalized, dissociated, and sorted and then recycled to the plasma membrane during Vg uptake [22]. In our study, we found that HlVgR was distributed throughout the cytoplasm of stage I and II oocytes (Fig. 3), suggesting that such oocytes are unlikely to take up Vg from hemolymph. In the stage III oocytes, positive signals were detected throughout the cytoplasm and in the external region of the oocytes. HlVgR was localized in the cytoplasm, especially in the peripheral region of the stage IV–V oocytes, which possess yolk granules in the cytoplasm [20].

Knockdown of *HlVgR* caused the delay or arrest of oogenesis in our study (Fig. 4), resulting in the inhibition of oocyte development from stage III to the subsequent stage. Control ticks started to lay eggs at 5 days after engorgement, whereas the oocytes of the RNAi-treated ticks at that timing were still developing to stage III. The ovaries of the RNAi-treated ticks showed an amberlite color, implying that Vg uptake in the oocytes starts from stage III. Seixas et al. [18] similarly reported that *RmVgR*-silenced female ticks showed a small degree of yolk uptake in the oocytes classified into the ovarian growth phase scores/indexes 2 and 3. They also showed a clear correlation between the amount of RmVgR mRNA and protein in the ovaries: RmVgR protein level was increased after the upregulation of mRNA expression in the ovaries of partially fed female ticks, and the level peaked before engorgement. Moreover, they described that when female ticks drop from the host animal, *VgR* mRNA expression levels decrease because the receptor is no longer important once Vg has already been taken up by the oocytes. However, the Vg concentration in the hemolymph reaches its highest level at 2–5 days after the beginning of oviposition in hard ticks [23–25]. As shown in our study, the expression level of *VgR* mRNA depends on the developmental stages of the oocytes. Therefore, the decrease of the *VgR* mRNA level was considered to be attributed to the decrease in the ratio of stage I and II oocytes to all oocytes. Oogenesis continues to progress throughout the oviposition period of around 1 month until the female ticks die.

Nurse or follicle cells, the providers of nutrients for insect oocytes, are non-existent in the ovary of ticks. Instead of such nurse or follicle cells, pedicel cells derived from ovarian wall epithelial cells are present in ticks. These pedicel cells are known to attach the oocytes to the ovarian wall [26, 27]. In addition to their structurally supportive role, it was speculated that tick pedicel cells have similar functions of nurse or follicle cells in insects. Ultrastructural observations led to the idea that yolk precursors, i.e. Vg, might be synthesized in pedicel cells of *A. triste* ticks and would then be transported into stage II oocytes [26]. In *D. melanogaster*, *VgR* mRNA is transported to the oocyte after synthesis in the nurse cells [21]. We found positive signals for *HlVgR* mRNA in both pedicel cells and the ovarian wall at the slow feeding, pre-oviposition, and oviposition periods (Fig. 2). *HlVgR* mRNA could be synthesized in the pedicel cells; however, further experiments are needed to clarify this point because HlVgR protein was not detected in pedicel cells and the ovarian wall by immunostaining.

## Conclusions

**Fig. 5:**
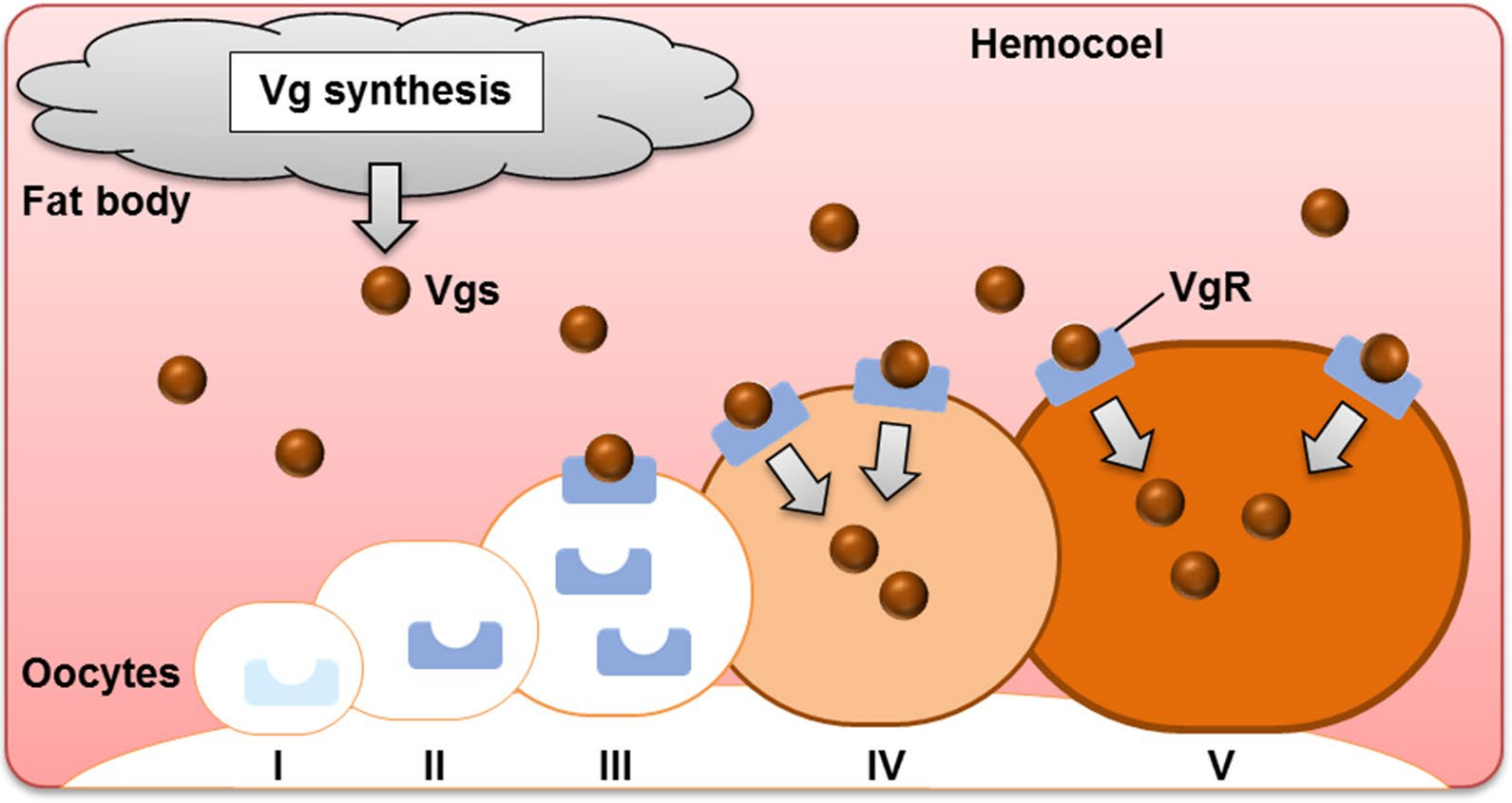
A schema of Vg uptake during oogenesis of parthenogenetic *H. longicornis*. Synthesized Vgs (Vg-2 and Vg-3) are released from the fat body into the hemocoel filled with hemolymph. The Vgs are taken up by oocytes of stages III, IV and V. *Abbreviations*: I, stage I oocyte; II, stage II oocyte; III, stage III oocyte; IV, stage IV oocyte; V, stage V oocyte; Vgs, vitellogenins; VgR, vitellogenin receptor

Our study revealed that an apparent shift in the intracellular localization of HlVgR mRNA and protein was associated with the developmental stages of oocytes in parthenogenetic *H. longicornis* ticks. This study showed that Vg uptake *via* VgR is a critical process for the transition of oocytes from stage III to IV in the tick. Taken together with findings from our previous studies [11–13, 17, 20, 28], we propose a model of Vg uptake during oogenesis in parthenogenetic *H. longicornis* (Fig. 5). To date, multiple Vgs (*HlVg-1*, *HlVg-2*, and *HlVg-3*) were identified from the parthenogenetic *H. longicornis* [28]. This study revealed that the *HlVg-1* transcript was detected only in the midgut of this tick species. The *HlVg-2* was expressed in the ovary and fat body, while the *HlVg-3* was found only in the fat body. “Vgs” in Fig. 5 includes HlVg-2 and HlVg-3 proteins based on our previous results. In other tick species, *DvVg1* mRNA was detected in the midgut, fat body, and ovary of *D. variabilis* [29, 30]. However, *DvVg2* mRNA was detected in both fat body and midgut but not found in the ovary. mRNA expression of *AhVg1*was observed in the fat body, midgut, and ovary, while *AhVg2* was detected in both fat body and midgut in *A. hebraeum* [31]. In *R. microplus*, Vg-1 and Vg-2 are synthesized in the ovary [32]. The contribution of each *Vg* to vitellogenesis remains unclear; however, ovarian molecules are considered as targets for anti-tick vaccine development as they can affect tick biology by decreasing oogenesis and embryogenesis [33]. After blood-feeding, immunoglobulins pass through the midgut epithelium and then arrive in the hemolymph of arthropods while retaining their immunological characters [34]. It is believed that cell membrane receptors serve as vaccine candidate molecules in blood-feeding arthropods. Further understanding of the molecular mechanisms regulating oogenesis will provide valuable information for the control of ticks. Moreover, our current findings may help to clarify the interaction between transovarially transmitted pathogens and ovarian molecules, especially Vg or VgR, in ticks. The potential of HlVgR as an antigen for the development of an anti-tick vaccine could induce infertility in ticks and thus inhibit the transmission of tick-borne diseases.

## Methods

### Ticks and animals

The parthenogenetic *H. longicornis* ticks (Okayama strain) were maintained at the National Research Center for Protozoan Diseases, Obihiro University of Agriculture and Veterinary Medicine, Obihiro, Japan. The female ticks were fed on Japanese white rabbits (Japan SLC, Shizuoka, Japan) as described previously [35]. Eight-week-old female BALB/c mice (CLEA Japan, Tokyo) were used to produce anti-HlVgR polyclonal antibody. The rabbits were reared in a temperature- and humidity-regulated room under controlled lighting, water and commercial regular chow. The mice were maintained in a temperature- and humidity-regulated P2 room under same conditions as rabbits.

### Reverse transcription and real-time PCR

For collection of the ovaries, 30 ticks at the unfed, 20 ticks at slow feeding (3 days after attachment), 5 ticks at rapid feeding (4–6 days after attachment), and 3 ticks at engorgement (5–7 days after attachment) periods were dissected. Twelve engorged females in individual glass tubes were placed in a container. The container was then placed in dark incubator at 25 °C and saturated humidity. The ovaries were also sampled from 4 ticks at 4 days (4dAE; pre-oviposition), 4 ticks at 7 days (0dAO; beginning of oviposition) and 4 ticks at 17 days (10dAO) after engorgement. Total RNA was extracted from the collected ovaries using TRI® reagent (Sigma-Aldrich, St. Louis, MO, USA) and incubated with DNase (TURBO DNA-*free*™ Kit; Life Technologies, Frederick, MD, USA) to remove genomic DNA. Single-strand cDNA was synthesized using purified total RNA and a ReverTra Ace® qPCR RT Kit (Toyobo, Osaka, Japan) according to a previous report [36]. Subsequently, real-time PCR was conducted using a 7300 Real-Time PCR System (Life Technologies) and THUNDERBIRD® SYBR qPCR Mix (Toyobo) according to the manufacturer’s protocol. The primers used in our study are listed in Table 2. Standard curves used for the calculation of relative gene expression were created from five-fold serial dilutions of cDNA derived from the ovaries of engorged females. The PCR conditions were as follows: 95 °C for 10 min, 40 cycles of denaturation at 95 °C for 15 s, and annealing/extension at 60 °C for 60 s. Dissociation curves for individual wells were created to confirm the single PCR product. Data were analyzed by the 7300 system SDS software version 1.4 for Windows 7. The control amplification was carried out using the primers designed for *H. longicornis* ribosomal protein *P0* (*HlP0*) (GenBank: EU048401). All reactions were run in duplicate, and data obtained were used to calculate the mean ± standard deviation (SD). The differences in relative gene expression levels of *HlVgR* between each sample were compared using the Student t-test (*vs* Unfed) or Welchʼs t-test (*vs* Engorgement), depending on the data distribution. Statistical significance was set at *P* < 0.01.

**Table 2:** Primers used in this study

Experiment	Description	Sequence (5′–3′)
Real-time PCR	HlP0-Fwd	CTCCATTGTCAACGGTCTCA
	HlP0-Rev	TCAGCCTCCTTGAAGGTGAT
	HlVgR-Fwd	GACCGTTCTTGCTTCTGTCC
	HlVgR-Rev	GCGACCATCACACCAGTAGA
*In situ* hybridization	T7-HlVgR-1-Fwd	TAATACGACTCACTATAGGCTGTCCCTCGGATGACTTCTCCTG
	HlVgR-1-Rev	CCCCACAGTCGATCATGCCA
	HlVgR-1-Fwd	CTGTCCCTCGGATGACTTCTCCTG
	SP6-HlVgR-1-Rev	ATTTAGGTGACACTATAGCCCCACAGTCGATCATGCC
Expression of recombinant HlVgR protein	XhoI-HlVgR-Fwd	CCGCTCGAGATCTACTGGTGTGATGGT
	EcoRI-HlVgR-Rev	GGAATTCACAGTGGCTGCTTTCGT

### Preparation of histological sections of ovary

Thirty-two ovaries in total collected at the same periods as described in the previous section were fixed with 4% paraformaldehyde-0.1% glutaraldehyde in phosphate-buffered saline (PBS) at 4 °C, overnight. The ovaries (10 µm thick) were sectioned with a cryostat (CM3050 S, Leica Biosystems, Wetzlar, Germany) as described previously [37]. To prepare paraffin sections, a total of 55 ovaries were immersed in 4% paraformaldehyde-2.5% glutaraldehyde in PBS at 4 °C, overnight. After washing with PBS, the ovaries were pre-embedded in 3% agarose (Sigma-Aldrich) in PBS and then embedded in paraffin (Sakura Finetek Japan, Tokyo, Japan). The sections (5 µm thick) were cut on a REM-700 microtome (Yamato Kohki Industrial, Saitama, Japan).

### *In situ* hybridization

*In situ* hybridization was carried out according to the manufacturer’s protocol of the *in situ* hybridization reagents starting kit (Nippon Gene, Tokyo, Japan). RNA probes were generated from PCR products with T7 or SP6 ends produced by amplification of ovary cDNA using gene-specific primers (Table 2). The PCR was performed by a GeneAmp® PCR System 9700 (Life Technologies) and PrimeSTAR® MAX DNA Polymerase (Takara Bio, Shiga, Japan). The cycling conditions were as follows: 30 cycles of denaturation at 98 °C for 10 s and annealing/extension at 68 °C for 60 s. The PCR products (534 bp) were purified using the QIAquick Gel Extraction Kit (Qiagen, Hilden, Germany). Sense and antisense RNA probes were generated using purified template DNA, T7, and SP6 RNA polymerase, respectively, and digoxigenin-11-UTP (Roche Diagnostics GmbH, Mannheim, Germany) for 60 min at 37 °C (for T7 RNA polymerase) and 40 °C (for SP6 RNA polymerase). Synthesized RNA probes were confirmed by electrophoresis on a 1.5% agarose gel and were then treated with alkali hydrolysis buffer for 44 min at 60 °C.

Paraffin sections of the ovaries were deparaffinized and treated with 5–8 µg/ml proteinase K (Nippon Gene) for 20 min at 37 °C. They were acetylated for 15 min at room temperature by adding 1 ml of acetic anhydride into 400 ml of acetylation buffer and then pre-hybridized in hybridization solution for 30 min at 42 °C. Hybridization was performed for 16 h at 42 °C after adding 1 µg/ml of sense or antisense probe per section. Hybridized sections were treated with blocking reagent (Roche Diagnostics GmbH) for 30 min at room temperature, treated with anti-digoxigenin-AP Fab fragments (Roche Diagnostics GmbH) for 1 h at room temperature, and then reacted with BCIP®/NBT liquid substrate system (Sigma-Aldrich) for 24 h at room temperature. Following staining of hybridized probes, nucleus staining was performed with 1% methyl green (Muto Pure Chemicals, Tokyo, Japan) for 2 min at room temperature. The sections were then observed under a BZ-9000 light microscope (Keyence, Osaka, Japan). To determine expression sites of *HlVgR* mRNA in the ovary, the developmental stages of each oocyte were classified into five stages as described previously [20].

### Expression of recombinant HlVgR protein and production of mouse anti-HlVgR polyclonal antibody

A partial fragment (936 bp) corresponding to second ligand-binding domain with class A repeats of *HlVgR* (GenBank: AB299015) [17] was amplified by PCR with the forward primer containing a recognition site for *Xho*I and the reverse primer with a recognition site for *Eco*RI (Table 2). The PCR was conducted by a GeneAmp® PCR System 9700 (Life Technologies) and PrimeSTAR® MAX DNA Polymerase (Takara Bio). The cycling conditions were as follows: 30 cycles of denaturation at 98 °C for 10 s and annealing/extension at 68 °C for 60 s. The PCR product (952 bp) containing partial *HlVgR* and restriction enzyme sites purified by Wizard® SV Gel and PCR Clean-up System (Promega, WI, USA) was inserted into the pCold™ ProS2 DNA plasmid vector (Takara Bio), which had been digested with *Xho*I and *Eco*RI, with a Ligation-Convenience Kit (Nippon Gene) according to the manufacturer’s protocol. Recombinant plasmids were transformed into ECOS™-competent *Escherichia coli* DH5α (Nippon Gene). After transformation, the plasmids were confirmed by sequencing and used to transform ECOS™-competent *E. coli* BL21 (Nippon Gene). Expression of recombinant histidine-tagged HlVgR (rHlVgR; approximately 60 kDa) was induced with 0.1 mM isopropyl-β-D (-)-thiogalactopyranoside at 15 °C overnight. The rHlVgR was solubilized with binding buffer (pH 8.0) containing 8 M urea. The purification of rHlVgR was performed by His GraviTrap (GE Healthcare, Chicago, IL, USA) using elution buffer (pH 8.0) containing 8M urea under the denaturing conditions. After concentration of the purified rHlVgR using Pierce® Concentrator 9K MWCO (Thermo Fisher Scientific, Waltham, MA, USA), urea in the rHlVgR solution was removed using Slide-A-Lyzer™ G2 Dialysis Cassette 7K MWCO (Thermo Fisher Scientific). Fifty micrograms of soluble rHlVgR or PBS in Freund’s complete adjuvant (Sigma-Aldrich) was intraperitoneally injected into 8-week-old female BALB/c mice. The same antigen or PBS in Freund’s incomplete adjuvant (Sigma-Aldrich) was repeatedly injected intraperitoneally into the mice on days 14, 28 and 42 after the first inoculation. At 7 days after the last immunization, sera against rHlVgR were collected from mice. Anti-rHlVgR IgG in the sera was purified using the Melon™ Gel IgG Spin Purification Kit (Thermo Fisher Scientific) and was stored at − 30 °C until use.

### Immunostaining

Frozen sections were incubated with Liberate Antibody Binding solution (Polysciences, Warrington, PA, USA) for 5 min at room temperature for antigen retrieval. After washing with PBS, the sections were blocked in 10% goat serum in PBS at 4 °C overnight. The sections were then incubated with anti-rHlVgR IgG that had been diluted in 5% goat serum in PBS (1:10) for 1 h at 37 °C. Subsequently, the sections were washed with PBS and then reacted with Alexa Fluor® 594 goat anti-mouse IgG (H + L) (1:4000; Life Technologies) for 1 h at 37 °C. The sections were then mounted in ProLong® Gold Antifade Mountant with DAPI (Life Technologies) after washing with PBS. Fluorescent signals were observed under a TCS SP5 confocal laser microscope (Leica Microsystems, Wetzlar, Germany), and the signals were displayed in yellow color to allow clearer detection of signals.

### Histological observation of ovary in *HlVgR*-RNAi ticks

A 631-bp *HlVgR* fragment was amplified by PCR using T7 forward (5’-TAA TAC GAC TCA CTA TAG GCT GTC CCT CGG ATG ACT TCT CCT G-3’) and T7 reverse (5’-TAA TAC GAC TCA CTA TAG GTG CAG TTG CTC TCG TCC CGG CCG-3’) primers as described previously [17]. The PCR products were purified as described above and were then used to generate double-stranded RNA (dsRNA) using the T7 RiboMax™ Express RNAi System (Promega). Thirty-five female ticks fixed on a glass slide with adhesive tape were injected with 0.5 μl of 2 μg/μl (1 μg/tick) of the dsRNA of *HlVgR* or firefly *luciferase* as described previously [12]. Injected ticks were kept at 25 °C for 18 h in an incubator and then fed on rabbits. Twenty and fifteen engorged ticks of control and *HlVgR* RNAi groups, respectively, were collected from the rabbits and their body weight was measured. Statistical significance was assessed by the Student t-test. The ticks were then incubated in a chamber at 25 °C for monitoring of oviposition. The female ticks were dissected to collect 6 ovaries at the engorgement (5–7 days after attachment) and 3 ovaries at 5 days after engorgement. The ovaries immersed in PBS were morphologically observed under a SZX16 stereomicroscope (Olympus, Tokyo, Japan) with a DP21 camera (Olympus). After observation, the ovaries were immersed into 4% paraformaldehyde-0.1% glutaraldehyde in PBS at 4 °C, overnight, and then subjected to the preparation of frozen sections as described above. The frozen sections were stained with hematoxylin and eosin (Muto Pure Chemicals). The stained sections were then observed under a BZ-9000 microscope (Keyence). Moreover, total RNA was purified from the remaining ticks in each group and used to synthesize cDNA as described above. The gene silencing of *HlVgR* was assessed by real-time PCR using a 7300 Real-Time PCR System (Life Technologies) and THUNDERBIRD® SYBR qPCR Mix (Toyobo) as described above. The *HlVgR* expression was normalized to the *HlP0* mRNA expression. The expressions for control group were set to 1.0, and relative expressions for *HlVgR* RNAi group were calculated. The data represented the mean ± SD of triplicate samples. The differences between control and *HlVgR* RNAi groups were compared using the Welchʼs t-test. Statistical significance was set at *P* < 0.01.

## Abbreviations

AhVgR: *Amblyomma hebraeum* vitellogenin receptor
dsRNA: double-stranded RNA
DvVgR: *Dermacentor variabilis* vitellogenin receptor
HlVgR: *Haemaphysalis longicornis* vitellogenin receptor
LDLR: low-density lipoprotein receptor
PBS: phosphate-buffered saline
RaVgR: *Rhipicephalus appendiculatus* vitellogenin receptor
RmVgR: *Rhipicephalus microplus* vitellogenin receptor
RNAi: RNA interference
SD: standard deviation
TOR: target of rapamycin
Vg: Vitellogenin
VgR: Vitellogenin receptor
4dAE: 4 days after engorgement
0dAO: the beginning of oviposition
10dAO: 10 days after the beginning of oviposition
